# Alcohol Metabolism and Cancer Risk

**Published:** 2007

**Authors:** Helmut K. Seitz, Peter Becker

**Keywords:** Alcohol and other drug (AOD) consumption, heavy drinking, chronic AOD use, ethanol metabolism, carcinogenesis, cancer, upper respiratory system cancer, oropharyngeal cancer, laryngeal cancer, aerodigestive tract cancer, esophageal cancer, liver cancer, colon cancer, colorectal cancer, breast cancer, acetaldehyde, alcohol dehydrogenase (ADH), aldehyde dehydrogenase (ALDH), genetic factors, cytochrome P450 2E1 (CYP2E1), reactive oxygen species

## Abstract

Chronic alcohol consumption increases the risk for cancer of the organs and tissues of the respiratory tract and the upper digestive tract (i.e., upper aerodigestive tract), liver, colon, rectum, and breast. Various factors may contribute to the development (i.e., pathogenesis) of alcohol-associated cancer, including the actions of acetaldehyde, the first and most toxic metabolite of alcohol metabolism. The main enzymes involved in alcohol and acetaldehyde metabolism are alcohol dehydrogenase (ADH) and aldehyde dehydrogenase (ALDH), which are encoded by multiple genes. Because some of these genes exist in several variants (i.e., are polymorphic), and the enzymes encoded by certain variants may result in elevated acetaldehyde levels, the presence of these variants may predispose to certain cancers. Several mechanisms may contribute to alcohol-related cancer development. Acetaldehyde itself is a cancer-causing substance in experimental animals and reacts with DNA to form cancer-promoting compounds. In addition, highly reactive, oxygen-containing molecules that are generated during certain pathways of alcohol metabolism can damage the DNA, thus also inducing tumor development. Together with other factors related to chronic alcohol consumption, these metabolism-related factors may increase tumor risk in chronic heavy drinkers.

Epidemiologic studies of the last decades have unequivocally identified chronic alcohol consumption as an important risk factor for the development (i.e., pathogenesis) of various types of cancers, including cancers of the organs and tissues of the respiratory tract and the upper digestive tract (i.e., upper aerodigestive tract), liver, colon or rectum (i.e., colorectum), and breast (for a review, see Bagnardi et al. 2001). For these types of cancer, the following associations with alcohol consumption have been found:

- The highest cancer risk associated with alcohol consumption is seen for the upper aerodigestive tract— that is, the oral cavity, throat (i.e., pharynx), voice box (i.e., larynx), and esophagus. Heavy drinking (i.e., consumption of more than 80 g alcohol, or more than five to six drinks, per day^1^), especially combined with smoking, increases the risk of developing these cancers by a factor of 50 or more, depending on the population studied (Pöschl and Seitz 2004).
- Alcohol-related liver cancer (i.e., hepatocellular carcinoma) primarily develops in people with liver cirrhosis resulting from chronic excessive alcohol use.
- The risk for alcohol-related colorectal and breast cancer is smaller than that for the upper aerodigestive tract cancer. However, because these types of cancer have a high prevalence in the Western world, alcohol likely is an important risk factor. One study (Longnecker 1994) calculated that 4 percent of all newly diagnosed breast cancer cases in the United States primarily result from alcohol consumption.

Overall, however, only a small percentage of chronic heavy drinkers develop certain types of cancer; moreover, some people develop cancer even at relative moderate daily alcohol consumption. These observations suggest that a genetic predisposition may influence cancer risk. At least part of this genetic predisposition may be related to alcohol metabolism because the rate of alcohol metabolism is genetically determined. Alcohol metabolism primarily involves three groups of enzymes (see Figure) (for more information on the pathways of alcohol metabolism, see *Alcohol Research & Health* Vol. 29, No. 4, “Alcohol Metabolism: Mechanisms of Action”):

- Alcohol dehydrogenase (ADH) enzymes that oxidize beverage alcohol (i.e., ethanol) to acetaldehyde.
- Aldehyde dehydrogenase (ALDH) enzymes that oxidize the acetaldehyde to acetate.
- Cytochrome P450 2E1 (CYP2E1), a protein that is part of the microsomal ethanol oxidizing system (MEOS) and is involved in alcohol metabolism primarily after chronic alcohol consumption.

For several of these enzymes more than one genetic variant exists as follows (for more information, see the article by Edenberg, p. 5):

- Two of seven genes encoding ADH enzymes (i.e., the *ADH1B* and *ADH1C* genes) show polymorphism—that is, they exist in variants (i.e., alleles) that differ in their activities, resulting in the generation of different quantities of acetaldehyde.
- For the ALDH2 enzyme, the most important enzyme in the metabolism of acetaldehyde to acetate, two alleles exist, one of which has a very low activity, resulting in acetaldehyde accumulation after alcohol consumption; this genetic variant is present in a large proportion of Japanese and other East Asian people.
- The degree to which CYP2E1 is inducible by chronic alcohol consumption varies among people, and the induction may be genetically determined.

This review discusses the role of alcohol metabolism in alcohol-associated cancer development (i.e., carcinogenesis^2^), focusing mainly on the contribution of acetaldehyde and on genetic risk factors leading to increased acetaldehyde levels, such as certain alleles of the genes encoding ADH1C and ALDH2. This article also briefly describes the role in carcinogenesis of CYP2E1 and of compounds generated during CYP2E1-mediated alcohol metabolism. For a discussion of other mechanisms involved in alcohol-associated carcinogenesis—such as malnutrition with vitamin deficiency, concomitant smoking, the presence of certain bacteria in the gastrointestinal tract (resulting from poor oral hygiene and diet), and underlying alcohol-related diseases—see the recent review article by Pöschl and Seitz (2004).

## Acetaldehyde—A Carcinogen

According to the International Agency for Research on Cancer (IARC) (1999), overwhelming evidence indicates that acetaldehyde should be classified as a carcinogen in experimental animals. For example, acetaldehyde inhalation in rats and hamsters results in cancer of the nasal mucosa and the larynx. Similarly, long-term administration of acetaldehyde in drinking water results in changes characterized by excessive cell growth of the mucosa cells of the upper digestive tract. These mucosal alterations are similar to those observed following chronic alcohol ingestion. Finally, acetaldehyde induces inflammation and transformation of the cells lining the windpipe (i.e., trachea), interferes with the normal reproduction of cells, and enhances cell injury of the gastrointestinal mucosa associated with excessive cell growth.

One of the pathways through which acetaldehyde promotes cancer formation is by interfering, through several mechanisms, with the copying (i.e., replication) of DNA that occurs when cells divide. For example, acetaldehyde has been shown to cause alterations ranging from the exchange of single DNA building blocks (i.e., point mutations) in certain genes to gross chromosomal alterations (Obe et al. 1986). Moreover, acetaldehyde impairs the process through which naturally occurring damage to the DNA is repaired by inhibiting an enzyme that is important for the repair of a certain type of DNA damage.

In addition to these mechanisms, acetaldehyde can interact with DNA building blocks to form new molecules (i.e., DNA adducts). These adducts may trigger replication errors and/or mutations in cancer-causing genes (i.e., oncogenes) or in genes that normally prevent cancer development (i.e., tumor suppressor genes). For example, a major stable DNA adduct called N2-ethyl-2’-deoxyguanosine (N2-Et-dG) can be incorporated efficiently into new DNA molecules during DNA replication. However, although this DNA adduct has been detected in human white blood cells and in rat liver after alcohol administration, there is relatively little evidence that it actually induces DNA mutations.

Most DNA adducts are formed only at relatively high acetaldehyde concentrations that are not normally found in the body. However, a class of compounds known as polyamines can facilitate the formation of one mutagenic DNA adduct at acetaldehyde concentrations found in the gastrointestinal tract (50 to 100 μM). Moreover, the polyamine spermidine (which is found in tissues with rapidly dividing cells, such as the gastrointestinal mucosa) may react directly with acetaldehyde to form a molecule called crotonaldehyde, which can bind to the DNA and cause mutations (Theravathu et al. 2005; Salaspuro et al. 2006). This conversion of acetaldehyde to crotonaldehyde in the presence of spermidine and other polyamines also can occur in the mouth and throat (i.e., oropharynx), an area that is lined by a mucosa that undergoes rapid cell division.

Acetaldehyde is found in the saliva, which can lead to an elevated risk of oropharyngeal cancer. Cancer risk increases with the amount of acetaldehyde generated in the saliva, and patients with oropharyngeal cancer have elevated acetaldehyde concentrations in their saliva (Jokelainen et al. 1996). Because acetaldehyde in saliva is derived primarily from alcohol metabolism, it is clear in this case that the alcohol-associated cancer risk increases with the amount of alcohol consumed. Furthermore, the activity of the enzymes that regulate acetaldehyde formation and degradation— that is, ADH and ALDH—influences the incidence of alcohol-related gastrointestinal tract cancer among regular or heavy alcohol consumers.

### Sources of Acetaldehyde

Most acetaldehyde in the body is generated during ethanol metabolism, when the ethanol is oxidized to acetaldehyde by ADH or CYP2E1. Another source of acetaldehyde is bacteria living in the gastrointestinal tract (see Figure). For example, in the absence of oxygen, ADH-containing bacteria in the mouth and stomach can convert carbohydrates to acetaldehyde and ethanol. Although the stomach itself is usually free of bacteria^3^ because it is highly acidic, some people suffer from an inflammation of the stomach that is characterized by insufficient stomach acid production and which can lead to the development of stomach cancer. In these patients, bacteria can grow in the stomach because not enough stomach acid is produced. If the patients consume sugar (i.e., glucose), these bacteria can produce small amounts of ethanol and acetaldehyde. More importantly, if these patients consume alcohol, acetaldehyde concentrations in the stomach increase 6.5-fold (Väkeväinen et al. 2002; Salaspuro et al. 2006).

In addition to the acetaldehyde generated by cellular enzymes or gastrointestinal bacteria, considerable amounts of acetaldehyde are present in certain alcoholic beverages (e.g., calvados [an apple brandy]) and in cigarette smoke.

## Role of ADH in Alcohol-or Acetaldehyde-Associated Carcinogenesis

Genetic linkage studies conducted in alcoholics have provided striking evidence that acetaldehyde plays a central role in alcohol-associated carcinogenesis. These studies found that people who accumulate acetaldehyde because they carry certain alleles of the genes encoding ADH or ALDH have an increased cancer risk (Yokoyama et al. 1998). There are at least seven types (i.e., isozymes) of human ADH that are encoded by seven genes. These isozymes are categorized into five different classes based on structural characteristics. Class I isozymes account for most of the alcohol metabolism. These are three isozymes known as alpha (α), beta (β), and gamma (γ), which are encoded by the *ADH1A*, *ADH1B*, and *ADH1C* genes, respectively. (For more information on the classification of ADH isozymes, see the article by Edenberg, p. 5.)

For both the *ADH1B* and the *ADH1C* genes, several alleles exist that result in differences in the activity of the ADH molecules they encode (e.g., the rate with which the ethanol is oxidized to acetaldehyde). For example, the ADH1B*2 allele encodes an enzyme that is approximately 40 times more active that the enzyme encoded by the ADH1B*1 allele. Similarly, the enzyme encoded by the ADH1C*1 allele is 2.5 times more active than the enzyme encoded by the ADH1C*2 allele (Bosron et al. 1993). People who carry the highly active ADH1B*2 allele rapidly convert ethanol to acetaldehyde. This leads to acetaldehyde accumulation following alcohol consumption and results in toxic side effects, such as a flushing syndrome with sweating, accelerated heart rate, nausea, and vomiting. These adverse symptoms exert a protective effect against acute and chronic alcohol consumption (i.e., people with this allele typically drink little or no alcohol) and also appear to protect against alcohol-associated cancer development. The ADH1B*2 allele rarely is found in Caucasians but occurs more frequently in Asian populations.

The effects of the different *ADH1C* alleles on alcohol metabolism and, consequently, on drinking levels and alcohol-related carcinogenesis, are more subtle. They can best be studied in Caucasian populations in which the highly active ADH1B*2 allele is rare. (Similarly, Caucasians rarely carry a certain variant of an ALDH-encoding gene that also results in high levels of acetaldehyde accumulation and which will be discussed later in this article.) Studies on the relationship between *ADH1C* alleles and cancer occurrence in Caucasians have led to in contradictory results (Brennan et al. 2004; Harty et al. 1997). Harty and colleagues (1997) compared the risk of oral cancer associated with various alcohol consumption levels in people who carried two copies of the more active ADH1C*1 allele (i.e., who were homozygous for that allele^4^) with the risk in people who carried only one copy of this allele (i.e., who were heterozygous) or were homozygous for the less active ADH1C*2 allele. The study found that people who consumed eight or more drinks per day and were homozygous for the more active ADH1C*1 allele had a 40-fold increased risk for oral cancer compared with nondrinkers. In contrast, people who consumed the same amount of alcohol but who were heterozygous or homozygous for the less active ADH1C*2 allele had only four- to seven-fold increased risk compared with nondrinkers. At lower levels of alcohol consumption, the difference in cancer risk between the various gene carriers was less striking. This is not surprising, however, because higher levels of alcohol consumption also result in production of more acetaldehyde, which then can exert its carcinogenic effect.

**Alcohol Consumption and Colorectal Cancer**

The epidemiologic data concerning the association between alcohol consumption and colorectal cancer are not as clear as those concerning cancers of the upper aerodigestive tract. Most studies, however, detected a positive correlation between chronic alcohol consumption and colorectal cancer. In 5 of 10 case–control studies and all prospective cohort studies that considered alcohol consumption, researchers found a positive trend with respect to dose response (see Cho et al. 2004). Thus, the analysis of eight pooled cohort studies showed a significant trend between increasing alcohol intake and the risk of colorectal cancer, with consumption of more than 45 g (or about three drinks) per day increasing the risk by 45 percent (Cho et al. 2004). Other studies investigated the association between alcohol consumption and the development of growths in the colon that precede, and may develop into, colon cancer (i.e., adenomatous polyps). In five of six studies in which the effect of alcohol on adenomatous polyps was investigated, such a correlation was observed. Alcohol also may influence the progression from an adenoma to a carcinoma and may favor the development of high-risk polyps or cancer among patients with adenomas (Seitz et al. 2006). In 1999, a consensus conference of the World Health Organization on Nutrition and Colorectal Cancer concluded that chronic alcohol ingestion, even at low daily intake (one to three drinks or 10 to 40 g per day) results in a 1.5- to 3.5-fold increase in risk of rectal cancer and a lesser increase in risk of colonic cancer in both sexes (Scheppach et al. 1999). This conclusion was confirmed at an International Agency for Research on Cancer meeting on alcohol and cancer (Baan et al. 2007).

Additional studies have confirmed an increased risk of oropharyngeal and laryngeal cancer in alcohol consumers with the ADH1C*1 allele (Coutelle et al. 1997). Other case– control studies, however, have not been able to confirm this association (Olshan et al. 2001; Brennan et al. 2004). The negative results of these studies may, at least in part, result from the fact that the alcohol intake of the participants was low and may not have led to sufficiently high acetaldehyde levels.

More recently, two studies determined *ADH1C* polymorphisms in more than 400 heavy drinkers (i.e., people who consumed more than 60 g alcohol, or more than four drinks, per day) with various cancers of the upper aerodigestive tract, liver, and breast. The data were compared with carefully matched control patients with alcohol-related diseases (e.g., cirrhosis of the liver, pancreatitis, and alcohol dependence) but without cancer (Homann et al. 2006; Coutelle et al. 2004). Cancer patients and control subjects were of similar age and had similar histories of alcohol consumption (i.e., amount and duration of drinking) and cigarette smoking. The studies found that significantly more patients with alcohol-related cancers had at least one ADH1C*1 allele, or were homozygous for ADH1C*1, than did patients with other alcohol-related diseases. Statistical analyses determined a significant association between ADH1C*1 allele frequency and rate of homozygosity and an increased risk for alcohol-related cancer (p < 0.001). Finally, people who were homozygous for ADH1C*1 had a relative risk of developing esophageal, liver, and head and neck cancers of 2.9, 3.6, and 2.2, respectively, compared with people homozygous for ADH1C*2.

Other studies found that people who are homozygous for the ADH1C*1 allele had significantly higher acetaldehyde levels in their saliva than did heterozygous people or people who are homozygous for the ADH1C*2 allele (Visapää et al. 2004), similar to people with an inactive *ALDH2* allele (which is discussed in the following section). As mentioned earlier, acetaldehyde levels in the saliva may be important for cancer development. Saliva rinses the mucosa of the upper aerodigestive tract, and any acetaldehyde in the saliva may be taken up by mucosal cells. Moreover, mucosal cells contain little of the ALDH2 enzyme and therefore cannot efficiently break down acetaldehyde. As a result, acetaldehyde may bind to proteins and DNA in the mucosal cells and may initiate carcinogenesis. The hypothesis that acetaldehyde in the saliva contributes to tumor development is supported by the observation that acetaldehyde-fed rats with intact salivary glands showed excessive proliferation of the upper gastrointestinal mucosa, similar to the changes observed following chronic alcohol consumption. When the glands were surgically removed (i.e., when the animals no longer produced saliva), however, this excessive cell proliferation disappeared (Pöschl and Seitz 2004).

Because the γ-ADH enzyme, which is encoded by *ADH1C*, also is found in the mucosa lining the colon, other investigators have studied the relationship between the various *ADH1C* alleles and the development of alcohol-related colorectal cancer. Several recent studies suggest that the ADH1C*1 allele can play an important role in the development of in alcohol-associated colon cancer (Tiemersma et al. 2003). However, other studies have not come to the same conclusion.

In summary, numerous studies suggest that *ADH1C* alleles that result in acetaldehyde accumulation in the cells can enhance a drinker’s risk of developing alcohol-related cancers in a variety of tissues.

## Role of ALDH in Alcohol-or Acetaldehyde-Associated Carcinogenesis

The main enzyme that breaks down acetaldehyde in the body is ALDH2. It is encoded by the *ALDH2* gene, for which there are two main alleles, ALDH2*1 and ALDH2*2. The ALDH2*2 allele is caused by a point mutation in the normal ALDH2*1 allele, resulting in an almost inactive ALDH enzyme. This allele does not occur in Caucasians and is only found among Asian people. For example, approximately 10 percent of the Japanese population are homozygous for ALDH2*2. Moreover, approximately 40 percent of the Asian population are heterozygous. People who are homozygous for ALDH2*2 have an extremely low ALDH activity; when these people drink alcohol, acetaldehyde accumulates and the “flushing syndrome” develops. These people do not tolerate alcohol at all and are therefore generally protected against developing alcoholism. People who carry only one copy of the ALDH2*2 allele (i.e., who are heterozygous) also have greatly reduced (i.e., less than 10 percent) ALDH2 activity. Nevertheless, they can consume alcohol and may even become heavy drinkers and alcoholics.

Several epidemiological studies have demonstrated that the risk of alcohol-associated cancer of the aerodigestive tract is significantly elevated in people with low ALDH2 activity, with a relative risk of 11.0 for oropharyngeal and laryngeal cancer and 12.5 for esophageal cancer (Yokoyama et al. 1998; Seitz et al. 2004). In addition, these people have a 50-fold-higher risk than people without the ALDH2*2 allele of simultaneously developing a second tumor at another site of the esophagus. Finally, the risk of colon cancer is increased by a factor of 3.4 in people with an ALDH2*2 allele.

As is the case with people who are homozygous for the highly active ADH1C*1 allele, people who have one ALDH2*2 allele have elevated acetaldehyde levels in their saliva after a moderate dose of alcohol (Väkeväinen et al. 2000). In fact, acetaldehyde levels are nine times higher in the saliva than in the blood in these people, suggesting that it is reduced ALDH activity in the salivary glands rather than in the blood that leads to acetaldehyde accumulation in the saliva. ALDH-deficient heavy drinkers therefore represent an exceptional human “knock-out” model for long-term acetaldehyde exposure (Salaspuro et al. 2006).^5^ Whenever they drink, people in this group are exposed to extremely high acetaldehyde concentrations in their saliva, which is associated with a strikingly increased cancer risk. The harmful effects of salivary acetaldehyde are exacerbated further by the previously mentioned fact that acetaldehyde can be converted to cancer-causing crotonaldehyde in the presence of polyamines, which are elevated in tissue already injured by the local action of alcohol, such as the mucosa of the upper aerodigestive tract.

## Role of Ethanol Metabolism by Gastrointestinal Bacteria in Alcohol-Related Carcinogenesis

Bacteria and other microorganisms (e.g., yeasts) throughout the digestive tract can metabolize alcohol. For example, microorganisms (e.g., bacteria and yeasts) normally found in the mouth oxidize alcohol to acetaldehyde in the saliva. In addition, fecal bacteria can metabolize alcohol in the colon. (Following alcohol ingestion, the alcohol concentration in the colon is comparable with that in the blood.) The capacity of oral and gastrointestinal microbes as well as mucosal enzymes to metabolize acetaldehyde, in contrast, is rather limited. As a result, acetaldehyde concentrations in the saliva and in the colon during and after alcohol consumption are 10 to 100 times higher than in the blood (Salaspuro et al. 2006).

### Role of Oral Bacteria

Several lines of evidence support the assumption that oral bacteria play a role in salivary acetaldehyde production:

- Salivary acetaldehyde concentrations can be reduced by 30 to 50 percent by rinsing the mouth with antiseptic mouthwash (which kills bacteria in the mouth) after alcohol consumption (Salaspuro et al. 2006).
- Certain risk factors for the development of oral cancer, such as poor dental and oral hygiene as well as tooth loss, all of which are associated with high levels of bacteria in the mouth, also are associated with increased acetaldehyde concentrations in the saliva following alcohol consumption.

Increasing alcohol consumption increases salivary acetaldehyde concentrations in a dose-dependent manner. Salivary acetaldehyde concentrations are significantly higher in alcoholic patients with head and neck cancer than in a control population.

Many drinkers also smoke, and smoking affects acetaldehyde generation in the saliva after alcohol consumption. For example, smokers have twice as much acetaldehyde in their saliva as nonsmokers, if they consume the same amounts of alcohol (Salaspuro et al. 2006). Smoking approximately 20 cigarettes daily increases in-vitro salivary acetaldehyde by about 50 percent following alcohol consumption. Smoking influences salivary acetaldehyde levels through two mechanisms. First, it increases the capacity of oral yeasts and bacteria to produce acetaldehyde from ethanol. Second, cigarette smoke itself contains considerable amounts of acetaldehyde that dissolve in the saliva during smoking (Salaspuro and Salaspuro 2004). The significant increase in salivary acetaldehyde concentration during drinking and active smoking may explain why the two habits have a synergistic effect on the risk for upper digestive tract cancer (Salaspuro et al. 2006).

### Role of Fecal Bacteria

Acetaldehyde also can be produced by fecal bacteria. In fact, of all the tissues in the body, the mucosa in the colon contains the greatest amount of acetaldehyde per gram of tissue following alcohol ingestion (Seitz et al. 1990). Animal studies with normal rats and rats that have no bacteria in their intestines (i.e., germ-free animals) have clearly demonstrated that this acetaldehyde is produced by fecal bacteria. These studies found that acetaldehyde production and excessive cell growth are significantly reduced in germ-free animals compared with normal rats (Seitz et al. 1990).

Acetaldehyde has toxic effects on the colon mucosa, resulting in excessive cell growth, including growth at abnormal sites in the colonic mucosa, which is associated with an increased cancer risk. These effects were initially observed in experimental animals but recently have been confirmed in humans (Seitz et al. 2006). Moreover, the alcohol-related excessive growth of the colonic mucosa is especially pronounced in older animals, possibly because the sensitivity of the colon mucosa to acetaldehyde increases with age. This may have practical implications, as age alone is a risk factor for colorectal cancer.

One of the mechanisms through which acetaldehyde may increase the risk of colon cancer may involve folic acid, a vitamin that is important for cell regeneration and which helps protect the DNA by promoting the production of a compound called *S*-adenosyl-methionine. However, only in the colon are acetaldehyde concentrations high enough to break down folic acid, thereby destroying its function. Therefore, this mechanism may explain why people who consume small amounts of folic acid and methionine and ingest more than 20 g alcohol per day have a seven-times increased risk for a certain type of colon cancer compared with people with high folic acid and methionine intake and low alcohol consumption (Giovannucci et al. 1995).

## Role of Ethanol Metabolism by CYP2E1 in Alcohol-Related Carcinogenesis

In addition to being oxidized by ADH, ethanol also can be metabolized by the microsomal ethanol oxidizing system (MEOS), whose key component is CYP2E1. This enzyme is found in the liver but also is present in the mucosa of the entire gastrointestinal tract. The MEOS normally accounts for only a small percentage of alcohol metabolism; after chronic alcohol consumption, however, the activity of CYP2E1 can be increased (i.e., induced) 10- to 20-fold. According to recent studies, this induction of CYP2E1 may occur at a daily dose of 40 g ethanol (i.e., approximately three drinks) and after 1 week of consumption (Oneta et al. 2002). Moreover, CYP2E1 activity returns to normal within a few days following withdrawal from alcohol.

Several alleles of the gene encoding CYP2E1 have been identified, and not every person exhibits the same degree of CYP2E1 induction. Thus, some people show substantial induction, whereas others show no induction at all (Oneta et al. 2002). The specific reasons for this difference are still unknown. Moreover, the studies conducted to date in a variety of populations on CYP2E1 polymorphism as a risk factor in alcohol-related carcinogenesis do not yet allow for final conclusions.

During alcohol metabolism by CYP2E1, highly reactive, oxygen-containing molecules known as reactive oxygen species (ROS) are generated that can damage proteins and DNA. Accordingly, induction of CYP2E1 activity is not only associated with increased acetaldehyde production but also with increased ROS production. For example, the CYP2E1 concentration in the liver is correlated with the generation of one type of ROS called hydroxyethyl radicals (Seitz and Stickel 2006).

One way through which ROS produced by CYP2E1 exert their harmful effects is by interacting with fat (i.e., lipid) molecules in the cell membrane in a process called lipid peroxidation. This process results in the generation of additional reactive molecules that are chemically related to acetaldehyde, especially malondialdehyde and 4-hydroxynonenal (4HNE). For example, 4HNE binds to DNA and generates adducts that can cause mutations (i.e., are mutagenic) and are carcinogenic (Seitz and Stickel 2006). The role of ROS in cancer development is underscored by the fact that in animal experiments, the administration of molecules such as vitamin E that can interact with and detoxify ROS (i.e., radical scavengers), inhibits chemical-induced carcinogenesis in the esophagus (for a review, see Seitz and Stickel 2006). 4HNE also may contribute to cancer development by causing mutations in a tumor suppressor gene called p53. The protein encoded by this gene helps prevent tumor development by inducing a type of programmed cell death (i.e., apoptosis) in cells that are damaged and which could become cancer cells. When 4HNE binds to p53, the damaged cell becomes more resistant to apoptosis, which gives it a growth advantage because it is not removed adequately.

In addition, CYP2E1 also activates a variety of procarcinogens—compounds that in the body can be converted into cancer-causing substances. Some of these procarcinogens are present in tobacco smoke and the normal diet (e.g., compounds known as polycyclic hydrocarbons, hydrazins, aflatoxins, and nitrosamines). However, the interactions between ethanol metabolism and procarcinogen metabolism are complex and may depend, among other factors, on the degree of CYP2E1 induction, the chemical structure of the procarcinogen, and the presence or absence of ethanol in the body during procarcinogen metabolism. These issues are reviewed elsewhere (Seitz and Osswald 1992).

## Summary

Alcohol metabolism is a major contributor to the increased risk of certain cancers that is associated with heavy alcohol consumption. A crucial factor in this process is acetaldehyde, the first metabolite generated during ethanol oxidation. Acetaldehyde is a carcinogen that can promote cancer development through multiple mechanisms, including interference with DNA replication, induction of DNA damage, and formation of DNA adducts. The relative contributions of these mechanisms, however, remain controversial.

Because of the potentially harmful effects of acetaldehyde, any condition that leads to elevated acetaldehyde levels in the body increases cancer risk. Accordingly, people who carry *ADH* alleles that encode ADH enzymes with high activity or *ALDH* alleles that encode ALDH enzymes with particularly low activity are at increased risk of developing alcohol-related cancer. Bacterial production of acetaldehyde, particularly in the mouth and colon, also contributes to elevated acetaldehyde levels after alcohol consumption and further exacerbates its detrimental effects.

Other pathways of alcohol metabolism, such as the one mediated by CYP2E1, also play a role in alcohol-related carcinogenesis, particularly after chronic heavy alcohol consumption, when CYP2E1 activity is induced. In this case, it is not only the acetaldehyde that causes the damage but also the ROS that are generated during the CYP2E1-mediated reaction. But as with ADH- and ALDH-mediated alcohol metabolism, not all people are equally susceptible to this pathway of alcohol-related cancer development because different CYP2E1 alleles result in different levels of CYP2E1 induction following chronic alcohol consumption.

## Figures and Tables

**Figure:**
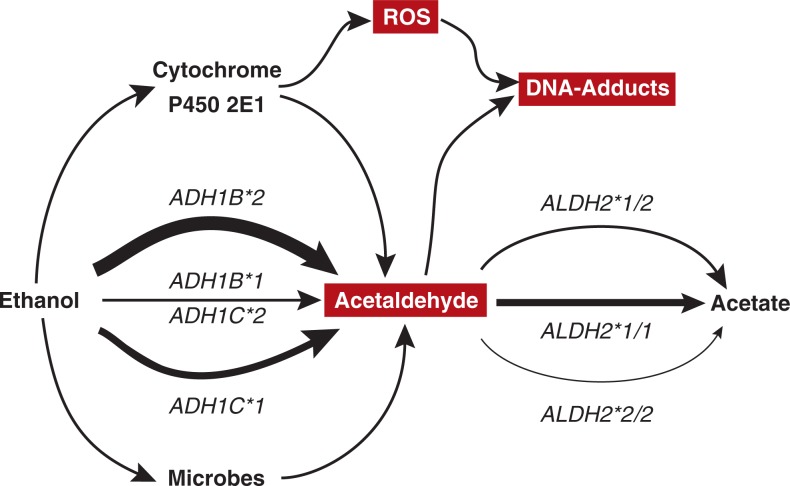
Pathways of ethanol metabolism and their role in carcinogenesis. Ethanol is oxidized to acetaldehyde through the actions of various alcohol dehydrogenase (ADH) enzymes (e.g., enzymes encoded by the *ADH1B* and *ADH1C* genes), through the microsomal enzyme cytochrome P450 2E1 (CYP2E1), and by microbes living in the human gastrointestinal tract (e.g., mouth and colon). The relative contributions of these pathways and the differences in activity between enzymes encoded by different *ADH1B* and *ADH1C* alleles is represented by the thickness of the arrows. Acetaldehyde is oxidized to acetate primarily by the enzyme aldehyde dehydrogenase 2 (ALDH2). Again, the thickness of the arrows indicates the rate of acetaldehyde oxidation in people carrying two active ALDH2*1 alleles, one active ALDH2*1 and one inactive ALDH2*2 allele, or two inactive ALDH2*2 alleles, respectively. Cancer-inducing substances (i.e., carcinogens) generated during the various pathways of alcohol metabolism are highlighted. These include acetaldehyde; highly reactive, oxygen-containing compounds (reactive oxygen species [ROS]) generated by CYP2E1; and adducts formed by the interactions of acetaldehyde or ROS with DNA.

## Footnotes

1 In the United States, a standard drink is frequently defined as the amount of beverage containing 0.5 ounces, or 14 grams, of pure alcohol. This amount is found in 12 fluid ounces (fl oz) of beer, 5 fl oz of wine, or 1.5 fl oz of 80-proof distilled spirits.

2 For a definition of this and other technical terms used in this article, see the glossary p. 32.

3 The exception to this statement are the *Helicobacter pylori* bacteria, which cause a large proportion of stomach ulcers.

4 Every person carries two copies of each gene, one inherited from the father and one inherited from the mother. These copies can be of the same allele or of different alleles.

5 This statement applies almost exclusively to Asians; because the *ALDH2*2* allele does not occur in Caucasians, this mechanism of acetaldehyde accumulation is irrelevant in this population subgroup.
