# Supplementary material for: Alcohol Metabolism and Cancer Risk
Source: Alcohol Res Health. 2007;30(1):38–47. (PMC3860434)
Supplement: Related texts

# Sub article: The Relationship Between Alcohol Metabolism, Estrogen Levels, and Breast Cancer Risk

**Published:** 2007

**Authors:** Helmut K. Seitz, Britta Maurer

Many epidemiological studies have demonstrated a positive association between alcohol consumption and an increased risk for breast cancer (for a recent review, see Hamajima et al. 2002). These studies have shown that even moderate alcohol consumption leads to a significantly increased risk for breast cancer. Moreover, Longnecker (1994) calculated that approximately 4 percent of newly diagnosed cases of breast cancer in the United States result from chronic alcohol intake.

Although the exact mechanisms through which alcohol exerts its cocarcinogenic effect on the breast remain unknown, a genetic predisposition may play an important role. This predisposition could involve the enzyme alcohol dehydrogenase (ADH), which breaks down alcohol to acetaldehyde. Recent studies have shown that people who carry genes encoding highly active ADH enzymes leading to elevated acetaldehyde levels are at particularly high risk for a variety of cancers, including breast cancer.

## ADH Variants and Breast Cancer Risk

As described in more detail in the accompanying article “Alcohol Metabolism and Cancer Risk,” there are seven types of ADH that are encoded by different genes. Moreover, two of the seven ADH genes, called *ADH1B* and *ADH1C*, are polymorphic—that is, they exist in more than one variant (i.e., allele). The enzymes encoded by these alleles differ in their activity and therefore result in the accumulation of different quantities of acetaldehyde. In Caucasians, polymorphism of the *ADH1C* gene is particularly relevant to cancer risk. This gene has two known alleles: a highly active allele called ADH1C*1 and a less active allele called ADH1C*2. Several case–control studies^1^ have assessed the relationship between the active ADH1C*1 allele (and, thus, elevated acetaldehyde levels) and the risk of breast cancer. Of these, three studies concluded that the ADH1C*1 allele plays a role in breast cancer development, particularly in women who have not yet entered menopause (i.e., are premenopausal); conversely, one study did not find such a positive correlation.

Freudenheim and colleagues (1999) compared 315 breast cancer patients and 356 age-matched control subjects. Among premenopausal (but not postmenopausal) women, the researchers found that breast cancer risk was higher in women carrying two copies of the ADH1C*1 allele (i.e., homozygous for ADH1C*1) compared with women carrying only one or no copy of the ADH1C*1 allele (i.e., heterozygous or homozygous for the ADH1C*2 allele). Moreover, premenopausal women who were homozygous for ADH1C*1 and had a higher level of alcohol intake^2^ were at greater risk of breast cancer than were comparable women with moderate alcohol intake (odds ratio 3.6, 95% CI 1.5–8.8).

More recently, Terry and colleagues (2006) compared more than 1,000 breast cancer patients with more than 1,100 control subjects. These researchers found that among women homozygous for ADH1C*1, a lifetime consumption of 15 to 30 g alcohol per day (which corresponds to approximately one to two drinks per day) was associated with a two-fold increase in breast cancer risk (95% CI 1.1–3.5). However, this increase in risk was not seen in women with the same alcohol consumption who were heterozygous or homozygous for ADH1C*2. Again, the increase in risk was particularly pronounced among premenopausal women. In a European study, Coutelle and colleagues (2004) reported that the ADH1C*1 allele was significantly more common in moderate alcohol consumers with breast cancer than in age-matched control subjects without cancer. Furthermore, women homozygous for ADH1C*1 had a 1.8-times greater risk of breast cancer than women with other allele combinations (95% CI 1.4–2.3).

In contrast to these three studies, Hines and colleagues (2000), who evaluated 465 breast cancer patients and 621 control subjects participating in the Nurses’ Health Study, did not find any effect of *ADH1C* polymorphism on breast cancer risk. However, this study included women with relatively low weekly alcohol consumption, which may not result in sufficiently high acetaldehyde concentrations.

It is important to note that the relationship between *ADH1C* polymorphism and cancer only can be studied in populations with significant alcohol consumption resulting in substantial acetaldehyde levels. Moreover, for accurately assessing the relationship between alcohol consumption, alcohol metabolism, and breast cancer, a substantial sample size is required.

## ADH and Estrogen Levels

One of the risk factors for breast cancer is an increased blood level of female sex hormones (i.e., estrogens), the most important of which is called estradiol. In some cases of breast cancer, the cancer cells carry docking molecules (i.e., receptors) for estrogens on their surface; these cancer cells depend on estrogen to grow. In other breast cancer cases, however, no estrogen receptors are found and these cells grow even without estrogen. Alcohol consumption and alcohol metabolism by ADH appear to affect the levels of estrogen and estrogen receptors, which may contribute to the alcohol–breast cancer association. However, this issue still needs to be fully elucidated. What currently is known is the following:

- The enzyme encoded by *ADH1C* not only metabolizes alcohol to acetaldehyde but also is involved in the metabolism of steroid hormones, including estrogens (McEvily et al. 1988).
- Some evidence suggests that alcohol enhances the expression of estrogen receptors in breast cells (Fan et al. 2000), which would increase breast cancer risk.
- Both in women with a normal menstrual cycle and in women taking oral contraceptives, blood concentrations of acetaldehyde after alcohol consumption were shown to be particularly high when estradiol levels reached their highest during the menstrual cycle (Eriksson et al. 1996). This could impact cancer risk because under these conditions the two risk factors acetaldehyde and estrogen would be combined.
- In premenopausal women, alcohol ingestion has been associated with higher estrogen concentrations in the blood, although some studies observed this effect only in women taking oral contraceptives (Singletary and Gapstur 2001).
- In another study of healthy premenopausal women, the administration of alcohol doses as low as 0.225 g pure alcohol per kg body weight (corresponding to approximately one drink) resulting in blood alcohol concentration not exceeding 25 mg per 100 mL (0.025 percent), caused an increase in serum estradiol concentrations of 27 to 38 percent while alcohol was detectable in the blood (Coutelle et al. 2004). This effect was observed during all phases of the menstrual cycle.
- For postmenopausal women, an increase of estrogens following alcohol consumption appears to depend on whether they use hormone replacement therapy (HRT) to treat menopausal symptoms. In women without HRT, estrogen increases were variable and depended on multiple factors. In women with HRT, the increase in estrogen levels was consistent and pronounced (Singletary and Gapstur 2001).

## Conclusions

Some of the published data suggest that acetaldehyde generated during alcohol metabolism may be involved in alcohol-associated development of cancer (i.e., carcinogenesis) in breast tissue, especially in women with higher alcohol consumption. If this evidence is confirmed, women with high levels of daily alcohol consumption who also are homozygous for the highly active ADH1C*1 allele may be at particularly high risk of developing breast cancer. In addition, there is some evidence that alcohol, even at low doses, increases serum estrogens under certain conditions and that this effect may be another important mechanism involved in alcohol’s cocarcinogenic actions.

## Footnotes

1 For a definition of this and other technical terms, see the glossary p. 32.

2 The group of women with higher alcohol intake was defined as including those women for whom the median of the reported average consumption over the past 20 years was 6.5 drinks per month or more for premenopausal women and 4.4 drinks per month or more for postmenopausal women.

## References

[b32-arh-30-1-38] Coutelle C, Höhn B, Benesova M (2004). Risk factors in alcohol-associated breast cancer: Alcohol dehydrogenase polymorphism and estrogens. International Journal of Oncology.

[b33-arh-30-1-38] Eriksson CJ, Fukunaga T, Sarkola (1996). Estrogen-related acetaldehyde elevation in women during alcohol intoxication. Alcoholism: Clinical and Experimental Research.

[b34-arh-30-1-38] Fan S, Meng Q, Gao B (2000). Alcohol stimulates estrogen receptor signaling in human breast cancer cell lines. Cancer Research.

[b35-arh-30-1-38] Freudenheim JL, Ambrosone CB, Moysich KB (1999). Alcohol dehydrogenase 3 genotype modification of the association of alcohol consumption with breast cancer risk. Cancer Causes and Control.

[b36-arh-30-1-38] Hamajima N, Hirose K, Tajima K (2002). Alcohol, tobacco and breast cancer: Collaborative reanalysis of individual data from 53 epidemiological studies, including 58515 women with breast cancer and 95067 women without the disease. British Journal of Cancer.

[b37-arh-30-1-38] Hines LM, Hankinson SE, Smith-Warner SA (2000). A prospective study of the effect of alcohol consumption and ADH3 genotype on plasma steroid hormone levels and breast cancer risk. Cancer Epidemiology, Biomarkers and Prevention.

[b38-arh-30-1-38] Longnecker MP (1994). Alcoholic beverage consumption in relation to risk of breast cancer: Meta-analysis and review. Cancer Causes and Control.

[b39-arh-30-1-38] McEvily AJ, Holmquist B, Auld DS, Vallee BL (1988). 3 Beta-hydroxy-5 beta-steroid dehydrogenase activity of human liver alcohol dehydrogenase is specific to gamma-subunits. Biochemistry.

[b40-arh-30-1-38] Singletary KW, Gapstur SM (2001). Alcohol and breast cancer: Review of epidemiologic and experimental evidence and potential mechanisms. JAMA: Journal of the American Medical Association.

[b41-arh-30-1-38] Terry MB, Gammon MD, Zhang FF (2006). ADH3 genotype, alcohol intake and breast cancer risk. Carcinogenesis.
